# Neuronal Intermediate Filament-Positive Paraneoplastic Neurological Syndrome in Cervical Squamous Cell Carcinoma

**DOI:** 10.7759/cureus.91866

**Published:** 2025-09-08

**Authors:** Anh-Thy P Nguyen, Bowon Joung, Adam Hagele, Kevin Bera, Bryan T Pham, Daniel I Kim

**Affiliations:** 1 Internal Medicine, University of California Riverside School of Medicine, Riverside, USA; 2 Hematology and Oncology, Loma Linda University Medical Center, Loma Linda, USA; 3 Internal Medicine, Loma Linda University Medical Center, Loma Linda, USA; 4 Department of Hematology and Oncology, Scripps Health, San Diego, USA; 5 Internal Medicine, University of California Riverside School of Medicine, Moreno Valley, USA

**Keywords:** cervical squamous cell carcinoma, gynecologic cancer, menorrhagia, neuronal intermediate filament, paraneoplastic syndrome

## Abstract

Paraneoplastic neurological syndromes (PNS) are a group of neurological manifestations of a variety of carcinomas that result from the generation of paraneoplastic antibodies (PAb) against the tumors that subsequently attack the central nervous system (CNS). The resultant damage from these onconeural antibodies leads to significant neurological manifestations and syndromes, which are sometimes seen with certain antibodies and tumors; however, the presentation of PNS is diverse. Diagnosis is often delayed due to a lack of clinical suspicion. Unfortunately, late recognition leads to worse clinical outcomes as PNS requires prompt treatment to prevent permanent neurological damage. Cases that are diagnosed early and receive immediate treatment have significantly improved outcomes and restoration of neurological function. Non-responders to treatment likely have irreversible neuronal damage at the time the syndrome was recognized. Surgical resection of the tumor is paramount in the treatment paradigm because this would eliminate the source of PAb generation. Immunomodulatory therapy further reduces ongoing damage to the nervous system.

We present an intriguing and the first documented case of PNS resulting from neuronal intermediate filament (NIF) antibodies detected in association with invasive cervical squamous cell carcinoma (SCC). The patient initially manifested with nonspecific weakness and loss of sensation that rapidly progressed to near complete loss of all motor function.

Given the diverse presentation of PNS cases, heightened awareness and a high index of clinical suspicion are crucial for early diagnosis and prevention of further neurological compromise.

## Introduction

Paraneoplastic neurological syndromes (PNS) are a rare group of immune-mediated neurological disorders triggered by an aberrant response to tumors. These syndromes arise due to the production of paraneoplastic antibodies (PAb), a subset of antibodies that target neural tissue and lead to paraneoplastic effects [1]. PNS commonly arise in association with lung, breast, gynecological, and prostate cancers. Although the exact mechanism of how malignancies lead to PNS is unclear, most cases of PNS are caused by immunologic mechanisms. The clinical presentation varies depending on which antibodies are produced and which organ system is affected [2]. There are limited cases of PNS associated with cervical cancer. The most recent case was reported in 2014, which details recurrent cervical cancer associated with Anti-Ma2 paraneoplastic encephalitis [3]. Even fewer studies demonstrate neuronal intermediate filament (NIF) antibodies in association with cervical cancer. NIF antibodies are significant because they are often associated with PNS. In a large study conducted by the Mayo Clinic Neuroimmunology lab, over 600,000 serum and cerebrospinal fluid (CSF) specimens from 65 patients undergoing workup for PNS between 1993 and 2017 were analyzed, and there were no cases associated with cervical cancer [4]. The limited number of PNS cases makes it difficult to create a standardized treatment plan; however, the overall treatment strategy revolves around three main pillars: tumor treatment, immunotherapy, and symptom management [5]. Our objective is to demonstrate that the gold standard treatment of PNS is early tumor resection combined with immunotherapy to prevent irreversible neurological decline [6].

## Case presentation

A 31-year-old previously healthy female with no prior medical history presented to the county safety-net hospital in March 2023 with acute onset oliguria and progressive muscle weakness. There was no history of recent fever, headache, abdominal pain, or hematochezia. No prior hematologic or neurocognitive abnormalities were noted. The patient was evaluated in February 2023 at a different institution for menorrhagia and acute flank pain. She was found to have severe anemia and renal impairment. She was unable to have regular outpatient visits for about six years prior to the start of these symptoms. Her gynecologic history was significant for irregular menses since she birthed her first child 12 years ago, and she denied having markedly heavy bleeding. On presentation to the outside hospital, her vaginal bleeding started that morning and was not reported to be very heavy. The gynecology team evaluated her menorrhagia with a transabdominal pelvic and transvaginal ultrasound, which were both normal. Furthermore, a computed tomography scan of the abdomen and pelvis (CTAP) and a renal ultrasound (US) ruled out urinary tract obstruction and hydronephrosis. She was also found to have acute kidney injury on chronic kidney disease given her atrophic kidneys, proteinuria and severe anemia. The gynecology team believed that her anemia was not explained entirely by her vaginal bleeding and attributed it to her stage 5 chronic kidney disease. The etiology of her renal disease was attributed to possible glomerulonephritis, and the patient was to follow up as an outpatient with nephrology. However, within 30 days of symptom onset, she developed acute renal failure with anuria, necessitating initiation of hemodialysis.

Following the new onset of renal failure, the patient exhibited rapidly progressive neurological deterioration including loss of the ability to eat, walk, and talk, despite preserved cognition. Physical examination revealed decreased muscle bulk and tone of all extremities. Workup for progressive weakness revealed positive intrinsic factor (IF) antibodies and elevated methylmalonic acid (MMA) levels. Serum ammonia was slightly elevated. Thyroid-stimulating hormone (TSH) and erythrocyte sedimentation rate (ESR) were within normal limits, but C-reactive protein (CRP) and serum folate were mildly elevated. Although aggressive intramuscular vitamin B12 supplementation was initiated, her neurologic symptoms persisted despite normalization of serum MMA levels. Serum studies for work-up of encephalopathy are shown in Table 1.

**Table 1:** Serum studies for encephalopathy work-up. TSH: thyroid-stimulating hormone, ESR: erythrocyte sedimentation rate, CRP: C-reactive protein

Serum Component	Patient Result	Reference Value
Methylmalonic acid (MMA)	815 nmol/L	87 - 318 nmol/L
Cobalamin (Vitamin B12)	777 pg/mL	254 - 1320 pg/mL
Ammonia	49 mcmol/L	25-32 mcmol/L
Folate	85.4 ng/mL	3.1 - 17.5 ng/mL
TSH	3.010 mIU/mL	0.358 - 3.740 mIU/mL
ESR	16 MM/hr	0-20 MM/hr
CRP	1.03 mg/dL	0 - 0.30 mg/dL

**Figure 1:**
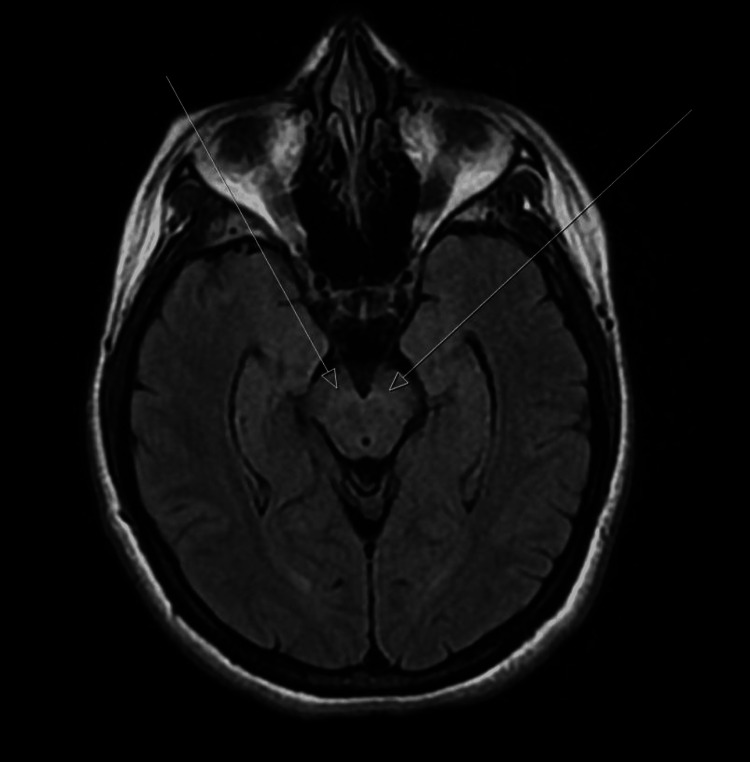
MRI brain showing symmetric T2 hyperintense signal abnormality within the midbrain between the red nuclei and cerebral peduncles (arrows), which is a sign of potential demyelinating disease.

**Figure 2:**
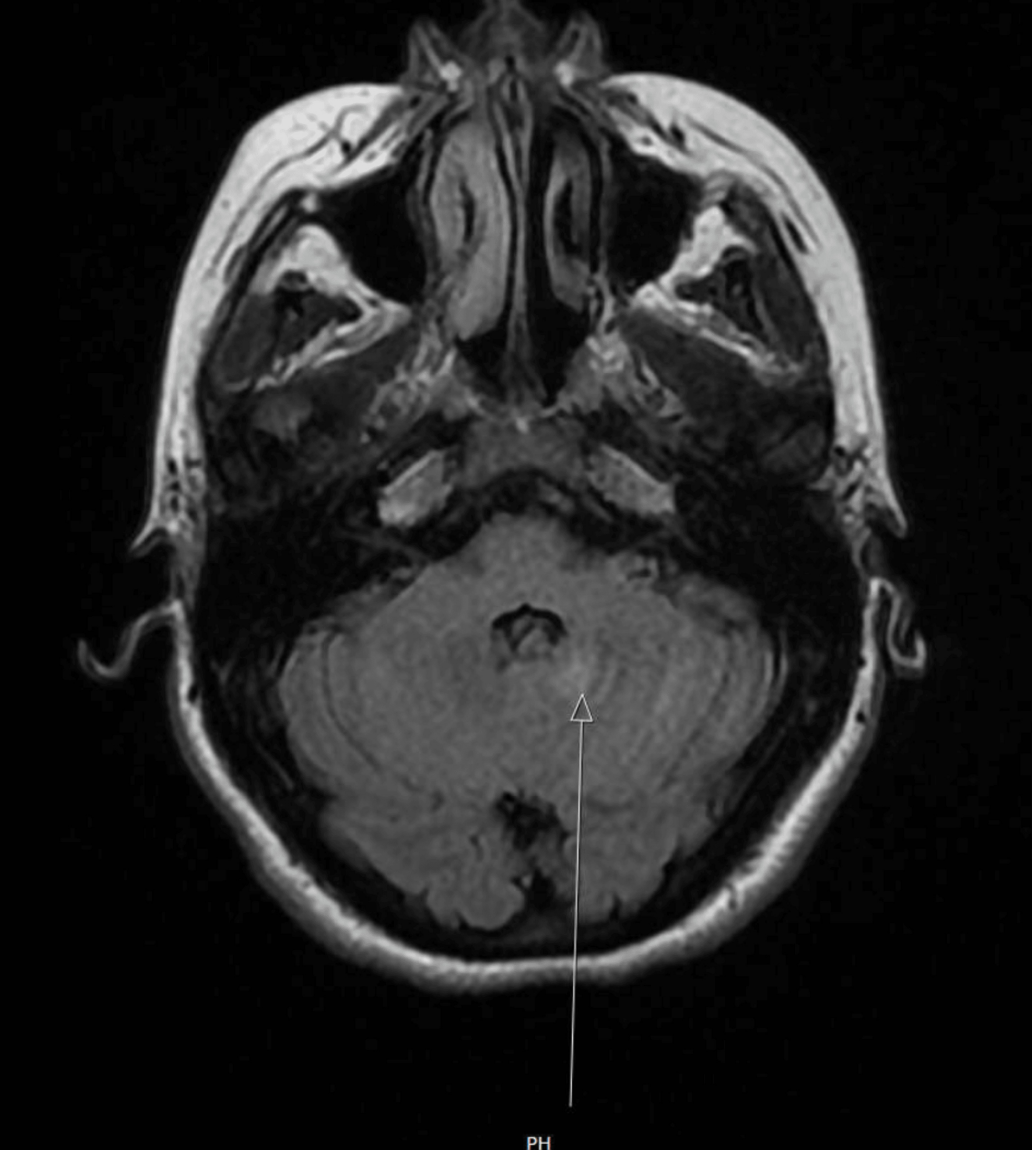
MRI brain demonstrating 1 cm focus of T2 hyperintense signal abnormality within the left cerebellar hemisphere, near the dentate nucleus (arrow), which increases suspicion for a possible demyelinating disease.

Magnetic resonance imaging (MRI) of the brain was obtained and showed signal abnormalities within the midbrain and near the dentate nucleus, which suggest possible demyelinating disease (Figure 1 and Figure 2). CSF studies were obtained via lumbar puncture (LP) with normal glucose, total protein and <5 WBC, indicating negative for infection, and negative cytology and oligoclonal bands. At this time, PNS screening labs were not obtained due to low suspicion for PNS as the etiology of her symptoms and evidence of potential demyelination. Due to the patient's history of menorrhagia, a repeat transabdominal pelvic ultrasound was completed in April 2023, which revealed a simple cyst in the right ovary and no acute changes.

**Figure 3:**
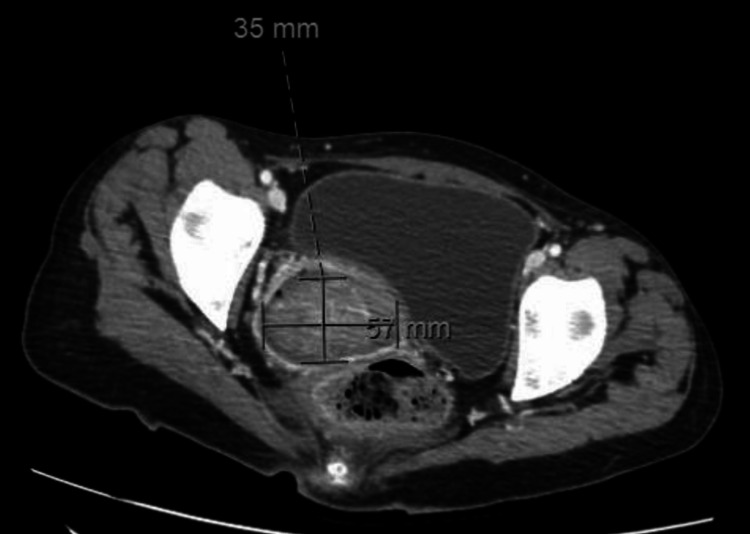
Computed tomography abdomen and pelvis (CTAP) showing complex mass, 5.7 x 3.5 cm hyperdense structure within the vaginal canal originating from the cervix, extending into the upper vaginal canal.

**Figure 4:**
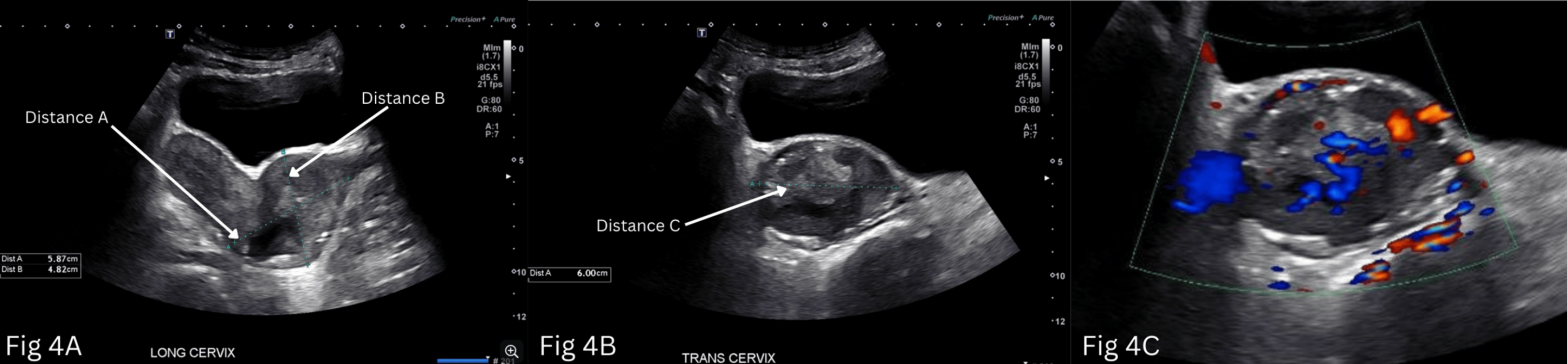
Transabdominal ultrasound in B mode showing longitudinal view (A) and transverse view (B) of the uterus. C. Transabdominal ultrasound of uterus in Doppler mode. (A) Longitudinal view: Distance A represents the length of 5.9 cm, Distance B represents width of 4.8 cm. (B) Transverse view: Distance C represents the height of 6 cm. Transabdominal ultrasound of uterus in Doppler mode (C) demonstrating areas of vascularity.

In the following weeks, the patient experienced recurrent abnormal vaginal bleeding, requiring multiple transfusions. A repeat CTAP in May 2023 revealed a new 5.6 x 3.5 cm hyperdense structure within the vaginal canal stemming from the cervix (Figure 3). Pelvic US and direct visualization confirmed a cervical mass (Figure 4), which was a poorly differentiated invasive squamous cell carcinoma (SCC) on biopsy. No other sources of malignancy were identified on whole body CT imaging. Given the new malignancy diagnosis, PNS was suspected, and repeat CSF studies were obtained. CSF results were negative for meningitis/encephalitis (Table 2), stiff person spectrum disorder (Table 3), and autoimmune encephalopathy (Table 4). However, CSF studies were positive for NIF-indirect immunofluorescence assay (IFA) titer and alpha-internexin cell-based assay (CBA) (Table 5). PNS studies were also sent at this time and were negative (Table 6).

**Table 2:** CSF Meningitis/Encephalitis Panel

Component	Patient Result
Cryptococcus neoformans/gattii	Not detected
Cytomegalovirus	Not detected
Enterovirus	Not detected
Escherichia coli K1	Not detected
Haemophilus influenzae	Not detected
Herpes simplex virus 1	Not detected
Herpes simplex virus 2	Not detected
Human herpesvirus 6	Not detected
Human parechovirus	Not detected
Listeria monocytogenes	Not detected
Neisseria meningitidis	Not detected
Streptococcus agalactiae	Not detected
Streptococcus pneumoniae	Not detected
Varicella zoster virus	Not detected

**Table 3:** Stiff Person Spectrum Disorders Serum Panel CBA: cell-based assay, Ab: antibody, LCBA: live cell-binding assay

Component	Patient Result	Reference Value
Amphiphysin Ab	Negative	Negative
DPPX Ab CBA	Negative	Negative
GAD65 Ab Assay	≤ 0.02	0.00
Glycine Alpha1 LCBA	Negative	Negative

**Table 4:** Neuromyelitis Optica (NMO) CSF Panel

Component	Patient Result	Reference Value
Aquaporin 4 Antibody, Cell-Based Assay, CSF	Negative	Negative
Myelin Oligodendrocyte Glycoprotein (MOG) Antibody, Cell-Based Assay, CSF	Negative	Negative

**Table 5:** Neuronal Intermediate Filament Indirect Immunofluorescence Assay (NIF IFA) Titer and Alpha-internexin Cell-Based Assay (CBA) of CSF

Result Name	Patient Result	Reference Value
NIF IFA Titer CSF	Positive - 1:64	< 1:2
Alpha Internexin CBA, CSF	Positive	Negative

**Table 6:** Paraneoplastic Autoantibody Evaluation, CSF

Test	Patient Result	Reference Range
anti-NR1	Negative	Not applicable
anti-LGI1	Negative	Not applicable
anti-CASPR2	Negative	Not applicable
anti-amphiphysin	Negative	CSF <1:1
anti-CV2	Negative	CSF <1:1
anti-Hu	Negative	CSF <1:1
anti-Ma and anti-Ta	Negative	CSF <1:1
anti-recoverin	Negative	CSF <1:1
anti-Ri	Negative	CSF <1:1
anti-Yo	Negative	CSF <1:1
anti-Zic4	Negative	CSF <1:1

The cervical tumor was staged as Ib3 vs occult stage IIb per National Comprehensive Cancer Network (NCCN) guidelines [7]. Thus, the treatment of choice was chemotherapy and radiation. On assessment by the gynecologic-oncology service, surgical resection was deemed infeasible despite high concern for PNS due to the patient’s poor functional status, irreversible neurological damage, and dialysis dependency. During her hospitalization in May, the patient’s mental status progressively deteriorated. She was unresponsive to verbal and painful stimuli and could not speak. She also developed tremors and bradykinesia that progressed to diffuse bilateral rigidity, hyperreflexia and clonus. Neurological physical exam findings were significant for her inability to open her mouth and risus sardonicus. The patient responded to questions by giving a thumbs up or squeezing fingers. Additional findings included horizontal nystagmus along with difficulty with tongue protrusion, drooling, and bowel incontinence.

Given her new diagnosis of cervical SCC, she was immediately treated for presumed PNS while awaiting CSF studies and hospital transfer for chemoradiation. Intravenous methylprednisolone, plasmapheresis (PLEX), and intravenous immunoglobulin (IVIG) were initiated given her barriers to tumor resection. Upon initiation of PLEX and IVIG, the patient showed slight improvement in her neurological status but without sustained significant cognitive improvement. Upon transfer to a cancer center, she received concurrent chemoradiation with dose-reduced cisplatin due to her comorbidities. Unfortunately, she acquired nosocomial pneumonia requiring IV antibiotics, which progressed to acute hypoxic respiratory failure requiring intubation. Due to intubation, infection, and continued need for dialysis, cisplatin was contraindicated due to risk for neutropenia. The patient was able to complete her planned course of radiotherapy with chemotherapy held.

Despite these treatments, she did not show significant improvement. Therefore, she was started on second-line anti-CD20 therapy with rituximab to further dampen her immune response to the tumor and reduce PAb generation. Despite all these treatments, the patient demonstrated minimal improvement in mental status, and her functional status worsened. After further discussion with the family about her likely irreversible neurological damage due to PNS, hospice was pursued.

## Discussion

PNS is a diagnosis of exclusion, requiring the systematic elimination of infectious, neurodegenerative and toxic or metabolic disturbances. Once these conditions were excluded, PNS was considered as a potential cause of a patient's progressive neurologic decline. The diagnostic criteria for PNS can be made based on the 2021 PNS-Care Score. Table 7 illustrates the score breakdown based on neurologic phenotype, presence of antibodies and timing of cancer detection after symptom onset [8]. A score of at least 8 confirms the PNS diagnosis. This patient’s neurologic presentation qualifies as high-risk because the combination of hyperreflexia, rigidity, weakness, bulbar signs, and tremor-like movements suggest clear brainstem and spinal cord signs, which fits the criteria of high-risk PNS per the 2021 PNS criteria. Furthermore, NIF antibody is arguably an intermediate-risk antibody as more research is demonstrating the association between NIF and PNS-associated cancer. Finally, gynecologic cancer was discovered less than two years after symptom onset. Based on these diagnostic guidelines, this case fits the diagnostic criteria for PNS with a score of 9.

**Table 7:** Paraneoplastic Neurological Syndrome Care Score Breakdown of Points Allocation Adapted from the 2021 Diagnostic Criteria for Neurologic Syndromes [8] under Creative Commons CC BY.

		Point Value
Phenotypes	High-Risk Phenotypes	3 points
	Intermediate-Risk Phenotypes	2 points
	Phenotype not associated with cancer	0 points
Antibody	High-Risk Antibody	3 points
	Intermediate-Risk Antibody	2 points
	Low-Risk Antibody / No Antibody	0 points
Cancer	Found	4 points
	Not found but follow up < 2 years	1 point
	Not found and follow up ≥ 2 years	0 points
Diagnostic Level		
Definite	≥ 8 points	
Probable	6 - 7 points	
Possible	4 - 5 points	
Non-PNS	≤ 3 points	

Advancements in biochemical technology have led to the identification of a variety of onconeural antibodies linked to specific tumor types and CNS phenotypes. Few gynecologic neoplasms such as ovarian teratoma, uterine or fallopian cancers are associated with PNS [9], and its association with cervical cancer is exceedingly rare. Even more uncommon is PNS linked to NIF autoimmunity, as observed in this case. NIF is a major cytoskeletal protein of neurons and glia that are part of neurological autoimmunity. In the central nervous system, alpha-internexin (aIN) is incorporated as a subunit. Anti-NIF has recently been identified as a novel biomarker of neuronal injury and other neurological diseases, such as PNS [10].

The diagnosis of NIF-PNS relies on CSF autoantibody testing using tissue-specific immunohistochemical or immunofluorescence staining techniques. While MRI brain and spine findings are often normal in early disease (therefore not specific for diagnosis), progressive cases can develop T2-weighted abnormalities in regions enriched with NIF subunits, such as the deep white matter, brainstem, cerebellum, and spinal cord, as seen in this patient [11]. Most commonly, anti-NIF antibodies are detected in neuroendocrine tumors, particularly small cell carcinoma, Merkel cell carcinoma, and, less commonly, hepatocellular carcinoma or T cell lymphoma. In current Western literature, there are only two reported cases of PNS associated with cervical cancer [3,12]. NIF-positive PNS typically presents with encephalitis, neuropathy, ataxia, and myelitis, all of which were exhibited in this case.

Early recognition of PNS-associated neurological symptoms is essential, as PNS often precedes malignancy in up to 80% of cases [13]. Oncologic treatment alone is often not sufficient for neurologic improvement because onconeural antibodies persist due to ongoing autoimmunity. Although the majority of onconeural antibodies respond poorly to immunotherapy, surface neuronal antibodies have shown greater sensitivity to immunotherapy [13]. Therefore, standard PNS therapy integrates both oncologic treatment and immunosuppressive therapies. In this case, the patient underwent chemoradiation and immunotherapy with rituximab, which resulted in slight improvement in neurologic status. However, there is a possibility that her condition could have improved significantly had the tumor been resected based on PNS cases in which early tumor resection and immunotherapy improved outcomes and prevented neurological decline [14].

In one trial, NIF-positive PNS secondary to small cell carcinoma, Merkel cell carcinoma or other neuroendocrine-lineage carcinomas treated with combinations of corticosteroids, rituximab, IVIG, PLEX, cyclophosphamide, cancer chemotherapy and/or azathioprine found 75% of patients treated had neurological improvements. Notably, patients with encephalopathy or myelopathy fully recovered [10]. Unfortunately, treatment options for this patient were constrained. The factor mainly contributing to the poor response is that we could not properly resect the tumor, and the treatment was limited due to her comorbidities. Therefore, the tumor was likely still secreting antibodies. Furthermore, it is unknown how long the tumor had been present before it was diagnosed, which makes it likely that the neurological damage was irreversible by the time immunomodulatory treatment was started. Although the patient underwent treatment with steroids, IVIG, and plasmapheresis with slight neurologic improvement, the response to treatment was suboptimal compared to the outcomes of the aforementioned studies.

The response in this case, unfortunately, is not similar to other cases of NIF-associated PNS due to the inability to completely resect and adequately treat the tumor. This case emphasizes the clinical importance of prompt diagnosis and treatment. We initially presumed that the patient's neurological deficits were due to vitamin deficiencies or electrolyte derangements. However, when her neurologic symptoms worsened despite adequate B12 levels, PNS was added to the differential because there was a continued and rapid decline in neurologic function. PNS was not initially considered in the differential due to its presumed rarity, and the treatment plan was not specific for PNS until two months after the patient’s initial presentation. It is possible that our patient’s refractory status was due to this delay in treatment that allowed the ongoing immunological damage or irreversible neuronal damage to continue. The goal of care moving forward was to prevent further neurological damage. Due to the ongoing inability to adequately control the secretion of these antibodies, the damage was likely prolonged, which highlights the importance of prompt recognition and treatment of the cancer.

## Conclusions

This first reported case of NIF-positive PNS associated with cervical SCC demonstrates the rapid progression of irreversible neurological damage in the absence of early intervention. The findings emphasize the critical need for early cancer detection and aggressive multimodal treatment, integrating oncologic therapy with immunosuppressive therapy. Timely diagnosis and intervention are paramount in preventing irreversible neuronal damage and improving patient outcomes in PNS. Additionally, it is important to consider PNS in atypical neurological presentations.

## References

[1] Eichmüller SB, Bazhin AV (2007). Onconeural versus paraneoplastic antigens?. Curr Med Chem.

[2] Viau M, Renaud MC, Grégoire J, Sebastianelli A, Plante M (2017). Paraneoplastic syndromes associated with gynecological cancers: a systematic review. Gynecol Oncol.

[3] Ney DE, Messersmith W, Behbakht K (2014). Anti-ma2 paraneoplastic encephalitis in association with recurrent cervical cancer. J Clin Neurol.

[4] Basal E, Zalewski N, Kryzer TJ (2018). Paraneoplastic neuronal intermediate filament autoimmunity. Neurology.

[5] Kerstens J, Titulaer MJ (2024). Overview of treatment strategies in paraneoplastic neurological syndromes. Handb Clin Neurol.

[6] Thapa B, Mahendraker N, Ramphul K (2025). Paraneoplastic syndromes. StatPearls [Internet].

[7] (2025). NCCN Clinical Practice Guidelines in Oncology. https://www.nccn.org/professionals/physician_gls/pdf/cervical.pdf.

[8] Graus F, Vogrig A, Muñiz-Castrillo S (2021). Updated diagnostic criteria for paraneoplastic neurologic syndromes. Neurol Neuroimmunol Neuroinflamm.

[9] Lin J, Wang M, Wang J, Li J (2022). Ovarian teratoma-related paraneoplastic neurological syndromes. Front Oncol.

[10] McKeon A, Shelly S, Zivelonghi C (2021). Neuronal intermediate filament IgGs in CSF: autoimmune axonopathy biomarkers. Ann Clin Transl Neurol.

[11] Rodriguez KM, Peguero E, Pina Y, Strosberg J, Verma N, Mokhtari S (2022). Neuronal intermediate filament (NIF) antibody positive paraneoplastic syndrome in a patient with metastatic neuroendocrine pancreatic carcinoma and treatment with ipilimumab/nivolumab. Neuro Oncol.

[12] Yucel D, Mekheal E, Kania B, Aron P, Kapoor A, Tailor RP, Maroules M (2022). A rare case of paraneoplastic syndrome of inappropriate secretion of antidiuretic hormone in cervical squamous cell carcinoma; a case report and literature review. J Community Hosp Intern Med Perspect.

[13] Darnell RB, Posner JB (2010). Breast, gynecologic, and testicular cancers. Paraneoplastic Syndromes.

[14] Devine MF, Kothapalli N, Elkhooly M, Dubey D (2021). Paraneoplastic neurological syndromes: clinical presentations and management. Ther Adv Neurol Disord.

